# DS-SIAUG: A Self-Training Approach Using a Disrupted Student Model for Enhanced Side-Scan Sonar Image Augmentation

**DOI:** 10.3390/s24155060

**Published:** 2024-08-05

**Authors:** Chengyang Peng, Shaohua Jin, Gang Bian, Yang Cui

**Affiliations:** Department of Oceanography and Hydrography, Dalian Naval Academy, Dalian 116018, China; 18908414801@163.com (C.P.); trighosts@163.com (G.B.); 13998435151@163.com (Y.C.)

**Keywords:** sample augmentation, side-scan sonar, self-training, underwater target detection, Disrupted Student

## Abstract

Side-scan sonar is a principal technique for subsea target detection, where the quantity of sonar images of seabed targets significantly influences the accuracy of intelligent target recognition. To expand the number of representative side-scan sonar target image samples, a novel augmentation method employing self-training with a Disrupted Student model is designed (DS-SIAUG). The process begins by inputting a dataset of side-scan sonar target images, followed by augmenting the samples through an adversarial network consisting of the DDPM (Denoising Diffusion Probabilistic Model) and the YOLO (You Only Look Once) detection model. Subsequently, the Disrupted Student model is used to filter out representative target images. These selected images are then reused as a new dataset to repeat the adversarial filtering process. Experimental results indicate that using the Disrupted Student model for selection achieves a target recognition accuracy comparable to manual selection, improving the accuracy of intelligent target recognition by approximately 5% over direct adversarial network augmentation.

## 1. Introduction

The detection and identification of underwater targets, such as shipwrecks, aircraft debris, pipelines, and reefs, are essential tasks in marine science research, resource exploration, and hydrographic surveying. These activities are critically important for maritime traffic safety, the development of marine fisheries, sonar detection, and military operations. Side-scan sonar is the primary technological means for subsea target detection due to its high-resolution imaging capabilities, making it the preferred choice for subsea target detection and identification [1,2,3,4,5]. Given the low efficiency, subjectivity, and experience dependency of manual recognition of side-scan sonar images, the use of machine learning algorithms for automated identification of these images has rapidly evolved [6,7,8,9,10,11,12,13,14,15]. Affected by the marine environment, side-scan sonar target images often contain a significant amount of noise, and the high cost of side-scan sonar target detection leads to a small dataset of high-quality target images, which severely impacts the accuracy and reliability of artificial intelligence in recognizing targets [16]. To augment the side-scan sonar target image dataset, Nayak et al. [17] utilized feature extraction and data enhancement techniques to recognize and augment image targets. However, the representativeness of the augmented samples was limited, impacting the model’s generalization ability. To utilize more image information, Nguyen et al. [15] attempted to increase the dataset through scattering, polarization, and geometric transformations. Although this improved the recognition rate of underwater human targets to 91.6%, the small data volume made the model prone to overfitting, limiting its generalizability. To fully utilize the background information of side-scan sonar images, style transfer methods were introduced to image augmentation [18,19]. Although this somewhat improved the recognition accuracy, issues with insufficient sample quantity and network model overfitting remained severe. Moreover, these methods mainly relied on the transformation and fusion of existing images, failing to generate images with entirely new features, thus limiting the transferability of sample diversity.

In recent years, deep generative models, such as Generative Adversarial Networks (GANs) [20], autoregressive models [21], regularized flows [22], and Variational Autoencoders (VAEs) [23], have shown tremendous potential in sample augmentation. Bore et al. [24] used conditional adversarial networks to simulate side-scan sonar images under specific conditions. However, this method was limited by seabed topography and sonar data, making implementation challenging. Jiang et al. [25] developed a semantic image synthesis model using adversarial networks, which rapidly generated new images from hand-drawn segmentation 0.5 mAPs and real side-scan sonar images, but the use of masks restricted the transfer of image styles and the generation of representative images. Denoising Diffusion Probabilistic Models (abbreviated as diffusion models) gradually corrected random noise through a reverse-simulated diffusion process to create new samples [26,27], but generating high-quality samples required multiple iterations [28,29]. To enhance efficiency, Yang et al. [30] proposed a rapid sample generation method for side-scan sonar using Denoising Diffusion Implicit Models (DDIM), also incorporating accelerated encoders to reduce sampling steps [30,31].

Although these methods can augment side-scan sonar images with entirely new features, there is still a lack of a stable model framework for the side-scan sonar field, and the quality of image generation still needs improvement. In particular, whether the augmented images are representative and can effectively improve the accuracy of underwater target recognition still require human involvement, severely affecting the efficiency of effective image selection. With the goal of target recognition, image generation models using self-training methods have made significant progress [32,33]. Given the current lack of a mature and stable algorithm model in side-scan sonar image augmentation, the problem of significantly affecting the accuracy of seabed target recognition due to limited sample sizes arises. This paper designs a side-scan sonar image augmentation method (DS-SIAUG) to address this issue. Based on the Denoising Diffusion Probabilistic Model (DDPM), the method combines this model with the YOLO recognition network for adversarial iterative training. It employs a Disrupted Student model to tag representative augmented images, reinputs these tagged images into the network, and repeatedly iterates this process to form a self-training side-scan sonar image generation model.

## 2. Materials and Methods

### 2.1. Overall Structure of DS-SIAUG

**Figure 1:**
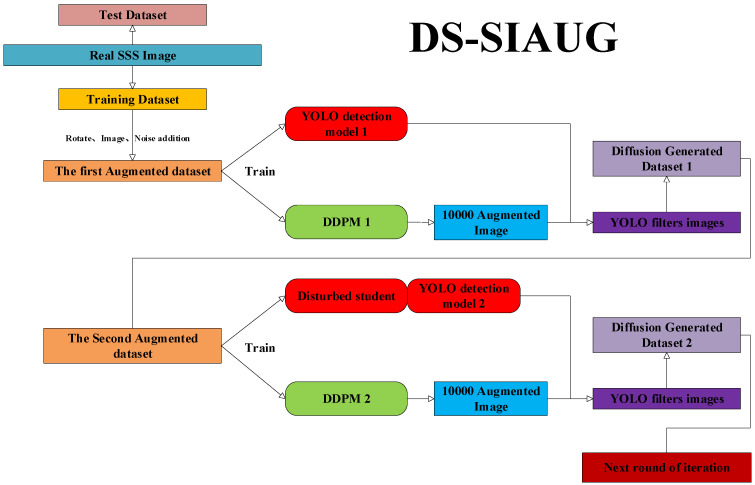
The overall training process of DS-SIAUG.

The overall training network structure of DS-SIAUG is illustrated in Figure 1. It comprises a DDPM diffusion generation model and a YOLO target detection model, with the Disrupted Student model incorporated into the training of the YOLO target detection model.

The DDPM diffusion module serves as an image generator, training to augment the side-scan sonar dataset. The YOLO network acts as a discriminator, detecting and determining the authenticity of the generated images. By designing an adversarial interaction between these two modules, we achieve the goal of augmenting the dataset, laying the foundation for subsequent target recognition tasks. The specific adversarial training process is as follows:

(1) Data preparation. Use the side-scan sonar shipwreck target as an example to explain the dataset augmentation method. Select a dataset of actual side-scan sonar shipwreck target images (Real SSS Image, 1,000 images), part of which is used as the test dataset (Test Dataset, 200 images) and part as the training dataset (Training Dataset, 800 images). To expand the training dataset, conventional image augmentation methods, such as rotation and mirroring, are first used to increase the training set to about 7000 images, called “The First Augmented Dataset”.
(2) Initial training phase. The First Augmented Dataset is input and trained using the DDPM (Deep Diffusion Model) and YOLOv5 network models. The result of this phase is obtaining the initial DDPM model (DDPM 1) and YOLO detection model (YOLO Detection Model 1).
(3) Image generation, filtering, and augmentation. Using the trained DDPM 1 model, generate a dataset of side-scan sonar images (Augmented Images, about 10,000 images) and input it into the YOLO Detection Model 1 for detection. A threshold is set (different thresholds will filter different numbers of side-scan sonar images with shipwreck features), with the threshold in this paper set at 0.5, filtering out about 2000 images (YOLO filter images). These filtered images are then augmented using conventional image augmentation methods, such as rotation and mirroring, increasing them to about 7000 images, called “Diffusion Generated Dataset 1”.
(4) Iterative training. Iterative training includes Disrupted Student training and diffusion model training. Disrupted Student training refers to adding student disruptions to “Diffusion Generated Dataset 1” and merging it with “The First Augmented Dataset” to train the YOLO detection model (YOLO Detection Model 2); diffusion model training refers to merging “Diffusion Generated Dataset 1” with “The First Augmented Dataset” to form “The Second Augmented Dataset”, which is then used to train the diffusion model (DDPM2).
(5) Using the DDPM2 model to regenerate the side-scan sonar image dataset (Augmented Images, about 10,000 images), input it into the YOLO Detection Model 2 for filtering, and perform conventional augmentation on the filtered dataset, naming the augmented dataset “Diffusion Generated Dataset 2”.
(6) Repeat steps (4) and (5), iteratively generating new datasets to continuously enhance the model’s detection capabilities.

In the above process, the Disrupted Student training of the diffusion dataset, the DDPM (Deep Diffusion Model), and the YOLOv5 detection model are the core modules of the adversarial reinforcement training structure.

### 2.2. Disrupted Student Training Model

**Figure 2:**
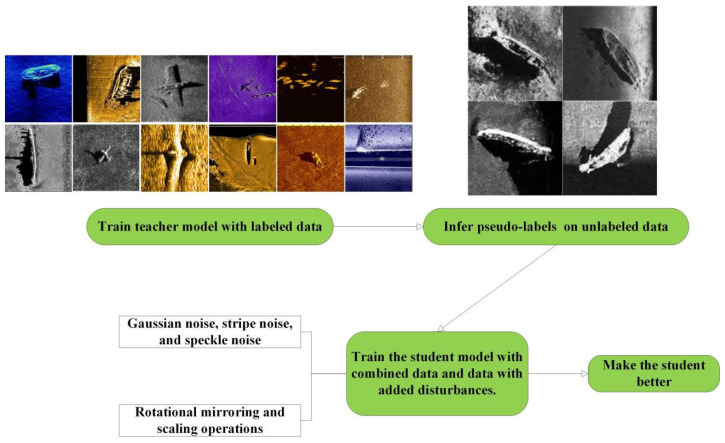
Schematic diagram of the Disrupted Student model structure.

The specific structure of the Disrupted Student model is shown in Figure 2.

This method can be considered an improved version of self-training, which itself is a method within semi-supervised learning (e.g., [34,35]) and knowledge distillation [36]. Initially, we use a teacher model to label the augmented images, and then disruptions (such as noise interference and geometric deformations) are added to these images. These labeled and disrupted images are then used to train the student model, ultimately aiming to achieve better performance than the teacher model.

Taking step four from Section 2.1 as an example, where the teacher model is the previously trained YOLO Detection Model 1 and the student model is the YOLO Detection Model 2 to be trained in this round, the steps are as follows:

First step: Training of the teacher model and automatic image labeling. The “First Augmented Dataset”, which has already been labeled, is used to train the teacher model, YOLO Detection Model 1. This mature teacher model then automatically labels the unlabeled “Diffusion Generated Dataset 1” with side-scan sonar shipwreck targets.

Second step: Adding disruptions to “Diffusion Generated Dataset 1”. We select some of the most common types of noise disruptions used in side-scan sonar and apply random rotations, mirroring, and deformations to the images to increase the difficulty of recognition for the student model.

Third step: The teacher model guides the student model’s training. The student model is trained using the disrupted “Diffusion Generated Dataset 1” along with the labels provided by the teacher model before the disruptions were added.

### 2.3. DDPM Model Structure

**Figure 3:**
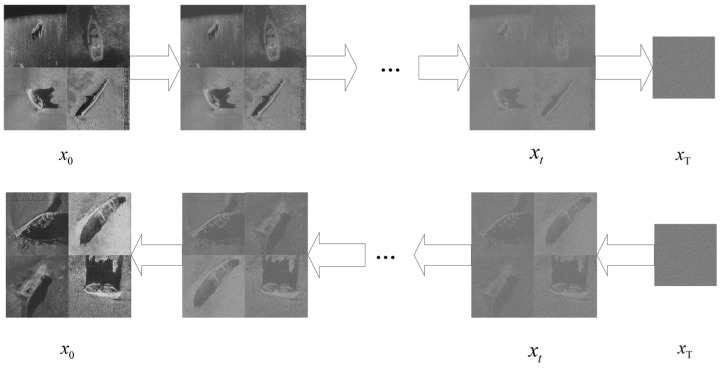
Forward and reverse diffusion processes of DDPM.

The diffusion model consists of two phases: “forward diffusion” and “reverse diffusion”. “Forward diffusion” refers to the process of repeatedly adding small “mist-like” effects (random noise) to a clear image, causing the image to gradually become blurred until it turns into an indistinguishable haze. “Reverse diffusion” is the inverse process of “forward diffusion”, where noise is progressively removed from this hazy image, restoring it to its original clarity. The diffusion model is trained and generates images through this process, as shown in Figure 3.

The diffusion model (DDPM) is defined in the form of a parameterized Gaussian Markov chain.

The “forward diffusion” process involves repeatedly adding a controlled amount of random noise to the image until it becomes “fogged”. It is assumed that the training data follow a distribution satisfying the $x_{0}\sim q(x)$ condition. The forward diffusion process adds Gaussian noise to the sample images in sequential time steps over *T* time steps, as shown in Equation (1).

(1) $$\begin{array}{l} q(x_{t}|x_{t-1})=N(x_{t};\sqrt{1-\beta_{t}}x_{t-1},\beta_{t}I)\\ q(x_{1:T}\left|x_{0}\right.)=\prod_{t=1}^{T}q(x_{t}\left|x_{t-1}\right.), \end{array}$$

In the equation, $q(x_{t}|x_{t-1})$ represents the conditional distribution, and $\beta_{t}$ is the variance of the Gaussian noise added at each step, which satisfies the condition $0<\beta_{1}<\beta_{2}<......\beta_{T}<1$. If *T* is sufficiently large, the diffused data $x_{T}$ lose the characteristics of the original data $x_{0}$, becoming random noise.

According to Equation (1), $x_{t}$ can be obtained through reparameterization sampling. Let

(2) $$\begin{array}{l} \alpha_{t}=1-\beta_{t}\\ {\bar{\alpha }}_{t}=\prod_{i=1}^{t}\alpha_{i}, \end{array}$$

After derivation, the relationship between $x_{t}$ and $x_{0}$ is obtained:

(3) $$q(x_{t}\left|x_{0}\right.)=N(x_{t};\sqrt{\alpha_{t}}x_{0},(1-{\bar{\alpha }}_{t})I),$$

The “reverse diffusion” model learns to remove noise. The training model progressively eliminates noise from random noise and gradually restores it to the initial image. This requires the model to be able to predict the appearance of the image after each denoising step. This process relies on a large amount of data and highly complex calculations; the model needs to learn how to accurately restore images at different noise levels.

If the reverse process is obtained,

(4) $$q(x_{t-1}\left|x_{t}\right.),$$

That is, by predicting the state of *t* − 1 from the state of *t*, we can gradually reconstruct an image from random noise $x_{T}$. DDPM uses a neural network to fit the reverse process.

(5) $$p_{\theta }(x_{t-1}\left|x_{t}\right.),$$

(6) $$q(x_{t-1}\left|x_{t},\right.x_{0})=N(x_{t-1}\left|{\tilde{\mu }}_{t}(x_{t},x_{0})\right.,{\tilde{\beta }}_{t}I),$$

Therefore, it can be derived that

(7) $$p(x_{t-1}\left|x_{t}\right.)=N(x_{t-1}\left|\mu_{\theta }(x_{t},t)\right.,\sum_{\theta }\left(x_{t},t\right)),$$

In the DDPM paper, the variance is not calculated; instead, the mean $\mu_{\theta }$ is fitted through the neural network to obtain $x_{t-1}$.

(8) $$\mu_{\theta }=\frac{1}{\sqrt{\alpha_{t}}}(x_{t}-\frac{1-\alpha_{t}}{\sqrt{1-\bar{\alpha_{t}}}}\varepsilon_{\theta }(x_{t},t)),$$

Because t and $x_{t}$ are known, the neural network only needs to fit $\varepsilon_{\theta }(x_{t},t)$.

The training process is as shown in Algorithm 1. First, extract a sample from the data and randomly select a time t from 1−T. Then, pass $x_{0}$ into the forward propagation process of Gaussian Diffusion, sample a random noise, and load it into $x_{0}$, forming $x_{t}$. Next, place $x_{t}$ and t into Unet. Unet generates a sine positional encoding based on time t and combines it with $x_{t}$, predicts the added noise, and returns it. Gaussian Diffusion calculates the loss between this noise and the random noise. Finally, calculate the L2 loss between the noise predicted by Unet and the previously sampled random noise through Gaussian Diffusion, compute the gradient, and update the weights.
**Algorithm 1 Training**1: **repeat**2: $x_{0}\sim q(x)$
3: t~Uniform({1,…,T})
4: ε~(0,I)
5: Take gradient descent step on $\nabla_{\theta }{\left\|\varepsilon -\varepsilon_{\theta }(\sqrt{{\bar{\alpha }}_{t}}X_{0}+\sqrt{1-{\bar{\alpha }}_{t}}\varepsilon ,t)\right\|}^{2}$
6: **until** converged

Repeat the above steps until the Unet network is fully trained.

Once the diffusion model is well-trained, the sampling process begins by sampling $x_{T}$ from the standard normal distribution. From t=T,…,1, the following steps are repeated sequentially. Sample z from the standard normal distribution as preparation for reparameterization, compute $\varepsilon_{\theta }$ based on the model, and sample z by combining $x_{t}$ and $x_{T}$, using reparameterization techniques to obtain $x_{t-1}$. After the loop ends, return $x_{0}$, which is the newly generated image. The specific training pseudocode is shown in Algorithm 2.
**Algorithm 2 Sampling**1: $x_{T}\sim N(0,I)$
2: **for** t=T,…,1 **do**3: z~N(0,I) if t>1, else z=0
4: $X_{t-1}=\frac{1}{\sqrt{\alpha_{t}}}(x_{t}-\frac{1-\alpha_{t}}{\sqrt{1-\bar{\alpha_{t}}}}\varepsilon_{\theta }(x_{t},t))+\sigma_{t}Z$
5: end for6: **return** $x_{0}$


### 2.4. YOLO Detection Model Structure

This paper selects the YOLOv5 detection model. As YOLO is a mature object detection network, this model achieves an optimal balance between efficiency and accuracy. Its well-designed input and feature extraction mechanisms, efficient feature fusion strategy, and flexible detection head configuration provide strong technical support for complex scenario side-scan sonar target detection. The YOLOv5 network is easy to deploy and has mature modules, making it the chosen network model for this study. The network structure of YOLOv5 mainly includes the following aspects:

(1) Input and Feature Extraction: YOLOv5 uses slicing technology to divide the input image into four feature maps with the same number of channels and reduces the number of parameters through concatenation operations across channel dimensions. After processing through scale and channel pathways, it further reduces the computational burden. A Spatial Pyramid Pooling (SPP) module at the end of this stage generates multi-scale feature maps, preparing for subsequent processing in the “neck” section. The input and feature extraction capabilities of the YOLOv5 network model effectively enhance computational efficiency and reduce model parameters, achieving improved detection speed and accuracy.
(2) Feature Fusion Layer: Utilizing the Path Aggregation Network (PANet), YOLOv5 efficiently merges feature maps of different scales in the feature fusion stage, enhancing the detection capability for targets of various sizes and effectively integrating shallow image features with deep semantic features, significantly improving detection performance.
(3) Detection Head: Composed of three convolutional modules, each process output features layers of three different scales capable of outputting predictions for five different categories and their location coordinates, plus a channel for representing confidence. This demonstrates YOLOv5’s high flexibility and accuracy in the field of object detection.

## 3. Experimental Validation

**Figure 4:**
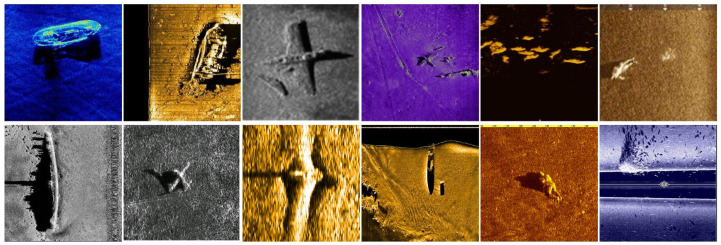
Dataset example.

The dataset selected for this study is gathered from various sources, including publicly available datasets, such as the shipwreck dataset released by Huoguanying. However, there are overlapping sections among these datasets, along with parts that were simply rotated for augmentation. Therefore, preprocessing of the dataset is necessary first. The final compiled dataset consists of approximately 1000 distinct and clear shipwreck target images. Additionally, the hardware used for model training includes Intel Xeon Silver 4410T*2* and *NVIDIA GeForce RTX 40904*. The software compilation environment includes PyTorch 1.6.0, CUDA 11.8, and Python 3.10 running on Windows 10. An example of part of the dataset is shown in Figure 4.

### 3.1. Adding Disruptions to the Student Model

Disruptions in the form of physical background noise and geometric deformations are added to the student model. The physical background noise includes Gaussian noise, stripe noise, and speckle noise, with specific additions detailed in Table 1. Geometric deformation disruptions involve rotating, mirroring, and scaling the images to alter the orientation of the ship’s body, with geometric disruption operations shown in Table 2. These measures enhance the student model’s ability to resist interference from two dimensions.

#### 3.1.1. Physical Background Noise

Gaussian Noise: Gaussian noise, also known as normal noise, is the most common type of noise. Its probability density function follows a Gaussian distribution. In side-scan sonar images, Gaussian noise typically originates from the thermal noise of electronic equipment and sensor reading errors, appearing as random fluctuations in grayscale values across the image. We randomly select 25% of the images to add Gaussian noise, with the noise intensity (i.e., the variance of the Gaussian noise) randomly varying between 0 and 0.5.

Stripe Noise: Stripe noise, or striping noise, often appears in side-scan sonar images as alternating light and dark stripes parallel to the scanning direction. This noise may be caused by the unevenness of the sonar equipment’s scanning mechanism, such as unstable mechanical movements of the scanning head or improper adjustments of electronic gain. Similarly, 25% of the images are randomly selected to add stripe noise, with the vertical gradient ratio randomly varying between 0 and 0.1.

Speckle Noise: Speckle noise is a type of multiplicative noise formed by the speckle pattern of multiple small beams converging. It is a common phenomenon in sonar images, particularly in high-resolution side-scan sonar images. Speckle noise makes the details on the image blurry, posing challenges to object edge recognition and classification. We choose 25% of the images to add speckle noise, with the noise size randomly selected between 0 and 0.1.

#### 3.1.2. Geometric Deformation Disruptions

Geometric deformation disruptions relate to operations commonly used in image processing, especially during data augmentation or image correction steps, and they include the following. Rotation: to simulate or correct the orientation of objects in different directions, images may be rotated by specific degrees. Although this operation can help improve the model’s generalization ability, it can also introduce noise, causing the original linear features and texture patterns to distort. We choose to rotate each image by 90, 180, and 270 degrees. Mirroring: mirroring transformation flips an image along a specific axis to simulate the symmetry of objects. This operation can increase data diversity in certain cases, but it may also alter the original spatial relationships and shape characteristics. We perform a mirroring operation on each image. Scaling: scaling operations, which magnify and reduce images, are used to simulate the observation effects of objects at different distances. However, scaling can introduce interpolation noise, particularly when enlarging, which may lead to artificial traces or distortion in the image. We apply random scaling to two-thirds of the images, choosing scaling ratios of 0.5, 0.75, 1.25, and 1.5 randomly.

### 3.2. Evaluation Metrics

Considering various aspects, such as clarity, feature diversity, and structural consistency, a variety of metrics are employed to assess the quality of generated images. The main metrics include the Fréchet Inception Distance (FID), Learned Perceptual Image Patch Similarity (LPIPS), Peak Signal-to-Noise Ratio (PSNR), Kernel Maximum Mean Discrepancy (MMD), and Structural Similarity (SSIM). FID measures the similarity between real and synthetic image sets in feature space by extracting features from both sets of images and modeling these features with Gaussian distributions to then calculate the distance between the means and covariances of the two Gaussian distributions. LPIPS evaluates perceptual differences between two images by learning the inverse mapping from synthetic to real images, thus forcing the generative model to reconstruct real images with a focus on perceptual similarity. PSNR evaluates similarity between two images by calculating the mean squared error and using the Peak Signal-to-Noise ratio to compare the original and generated images. MMD evaluates the similarity of feature distributions by mapping image sets to a kernel space using a fixed kernel function and calculating the mean difference between the two distributions. SSIM defines the perspective of image structural information, considering distortions in luminance, contrast, and structure as the three main factors affecting image quality, and bases its quality assessment on these factors.

### 3.3. DDPM Augmentation Effect Analysis

In the training process of the diffusion model, the size of the images has a decisive impact on the quality of the final generated images. The larger the image size, the more resources required for model training, as it involves processing a larger dataset to capture more detail. To explore the impact of image size on the quality of generated images, this study sets up three different sizes of generated images, 128 × 128(T1), 256 × 256(T2), and 512 × 512(T3), assessing the generation effects of different sizes during the training process. During training, the model’s state is saved every 10 iterations, and training parameters are adjusted according to different sizes to suit their respective dataset categories. Detailed training parameter settings can be seen in Table 3. Through this strategy, this study aims to provide directional guidance for optimizing generation tasks by thoroughly analyzing the impact of image size on the diffusion model’s ability to generate high-quality images.

**Figure 5:**
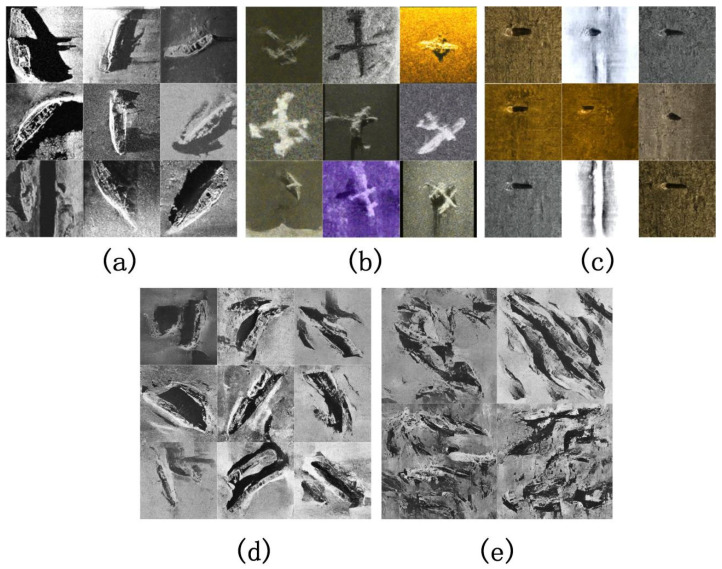
Generated image results. (**a**) A generated 128 × 128 image of a shipwreck. (**b**) A generated 128 × 128 image of an aircraft wreckage. (**c**) A generated 128 × 128 image of an underwater reef. Due to the limited data on aircraft wreckages and underwater reefs, higher-resolution images were not generated. (**d**) A generated 256 × 256 image of a shipwreck. (**e**) A generated 512 × 512 image of a shipwreck.

The image generation results using the aforementioned sizes are shown in Figure 5, featuring generated content including shipwrecks, aircraft wrecks, and underwater reefs.

**Figure 6:**
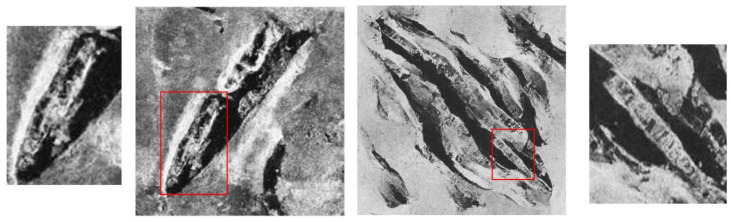
On the left is the generated image at 256 × 256 resolution, and on the right is the generated image at 512 × 512 resolution.

In the analysis of Figure 5a, we find that even at the smaller size of 128 × 128, the generated images still exhibit a clear resemblance to the original images. For example, in the generated shipwreck images, not only do the structure and form highly match the actual side-scan sonar images of shipwrecks, but they also capture shipwrecks of different sizes and positions, including the corresponding target shadows and texture details, which are closely linked to the characteristics of side-scan sonar imaging. In the case of Figure 5b, the contours and structure of the airplane are clearly defined, accurately depicting the airplane’s shape, and the background also displays rich topographical and geomorphological information, closely matching the features of airplane wreckages seen in side-scan sonar images. For the standalone reef in Figure 5c, its contours and shadows are clear, consistent with common reef formations, and some images even accurately reproduce the striping features of side-scan sonar images. For the high-resolution samples in Figure 5d,e, although some structural details of the shipwrecks appear fragmented as the resolution increases—which may be due to the lower resolution of the original training images, causing incoherence in the ship structures at higher resolutions—as shown in Figure 6, even when structural fragmentation occurs, the texture details of the shipwrecks still adhere to the characteristics of side-scan sonar imaging, demonstrating the model’s ability to capture detailed features.

From Figure 6, it can be seen that in the 128 × 128 images, the overall structure and hull outline of the shipwrecks are more complete and clear compared to the images of sizes 256 × 256 and 512 × 512, and they are extremely close to the real side-scan sonar images of shipwrecks. These images closely adhere to the principles of side-scan sonar imaging in terms of the geometric relationship between the target and its shadows. Simultaneously, the background texture and noise level also very closely match actual side-scan sonar images, displaying a diverse visual style. This indicates that the model can efficiently generate high-quality images that comply with the characteristics of side-scan sonar imaging and meet visual quality standards.

Through three comparative experiments conducted on generated images of different types and sizes, metrics, such as FID, LPIPS, PSNR, MMD, and SSIM, were calculated to assess image quality. An SSIM closer to 1 and a higher PSNR indicate better image quality, while lower values of FID, LPIPS, and MMD suggest higher image similarity. The experimental results are summarized in Table 4 showcasing the model’s performance in generating images of various types and sizes.

Based on the data from Table 4, the T1 model, as a smaller-sized generative model, demonstrated exceptional generative capabilities. For structurally simpler images of underwater rocks and aircraft wrecks, the distance between real and generated images in feature space is small, reflecting high-quality generation results. This finding is also reflected in the MMD index, where the score decreased as the size of the generated images increased, indicating that larger models have potential in simulating the feature distribution of real data, although achieving this may require longer training times. The PSNR assessment further emphasizes the T1 model’s efficiency in processing images of underwater rocks and aircraft wrecks. Meanwhile, the scores for the underwater shipwreck category indicate that the generated images are highly consistent in quality with the real images. The SSIM scores are lower in categories other than underwater reefs, indicating that there are certain differences in Structural Similarity between the generated images and the real images. Additionally, as the resolution of the generated images increases, the structural distortion of the generated images becomes more pronounced. This results in differences in the SSIM metric, meaning that the higher the resolution of the generated images, the more fragmented the images become, and the lower the SSIM score. The closeness and values below 1 of the LPIPS scores indicate that the generated images maintain a certain level of perceptual similarity to the original images, aligning with human visual perception.

### 3.4. Interference Experiments and Iterative Training Effects

Based on the experimental results from Section 3.3 and considering the current status of our dataset, we chose the 128 × 128 underwater shipwreck targets as the subjects for our experiments. According to the training procedure described in this paper, iterative training was conducted, and the entire iterative process lasted four rounds (the optimal performance was already achieved during the third round of iterative training).

#### 3.4.1. The Impact of Different Disruptions on the Student Model

To explore the performance capabilities of the student model under different disruption conditions, the Diffusion Generated Dataset was subjected to two types of disruptions: physical background noises, such as Gaussian noise and stripe noise, and geometric disruptions, such as scaling, rotation, and mirroring. The specific disruption conditions are shown in Table 5. The dataset without any disruptions is labeled C1, the dataset with added physical noise is labeled C2, the dataset with added geometric disruptions is labeled C3, and the dataset with both geometric and physical background noise disruptions is labeled C4.

After training with added noise, the performance of the student models in each group is shown in Table 6.

From Table 6, it is evident that when no disruptions are applied to the diffusion dataset (C1), the precision accuracy, recall rate, and mAP values are the lowest. The introduction of either physical background noise (C2) or geometric disruptions (C3) enhances the model’s ability to recognize targets, indicating that both types of disruptions somewhat improve the model’s generalization capacity. Comparatively, geometric deformation disruptions have a more significant impact on enhancing the model’s generalization ability and adaptability to complex scenarios. The simultaneous addition of physical background noise and geometric disruptions (C4) displays optimal performance, especially in terms of mAP values, suggesting that integrating various types of disruptions can further enhance the model’s robustness and performance.

In summary, adding disruptions (whether physical noise or geometric deformations) can enhance the model’s performance to a certain extent, with geometric deformation disruptions showing more significant effects. The combined disruption performs best primarily because the model needs to learn more complex features to adapt to a diverse data representation, thereby improving generalization capability.

#### 3.4.2. Iterative Training Effects (Compared with No Noise Filtering)

Based on the experimental results from Section 3.4.1, the optimal combined disruption mode is used for iterative self-training. A control experiment approach is employed where, for the T group, no disruptions are used for image filtering, and, for the G group, the combined disruption mode (physical noise + geometric deformation) assists the model in performing filtering. After four iterations, the related detection metrics after each iteration are shown in Table 7.

Horizontal Comparison (Intra-group Analysis)

T Group (Control Group): As the number of iterations increased, the overall performance of the T group showed an upward trend, particularly from T1 to T2. However, there was a performance decline from T3 to T4, which may indicate that the T group encountered a bottleneck in precision improvement with deeper iterations.

G Group (Combined Disruption Group): The G group exhibited significant performance improvements throughout the iterations. From G1 to G3, metrics, such as precision, recall, and mAP, consistently showed a steady increase. However, a decline in performance was observed in the G4 group, which could be due to the limited dataset where the model had already fully learned the features of the original dataset. This highlights the positive impact of combined disruptions on model performance in iterative self-training.

Vertical Comparison (Inter-group Analysis)

Initial Iteration (T1 vs. G1): In the first iteration, the G group performed significantly better than the T group across all metrics. This may be due to the introduction of combined disruptions, which enabled the G group’s model to learn more robust and generalizable feature representations, thereby performing better in the face of complex or unseen data. Subsequent Iterations (T2-T4 vs. G2-G4): In later iterations, the performance of the G group continued to surpass that of the T group, especially with more pronounced improvements in recall and mAP. This further demonstrates the effectiveness of the combined disruption mode in enhancing the model’s generalization ability.

Analysis of the Superior Performance of the G Group: Data Diversity: The combined disruptions (physical noise + geometric deformation) greatly increased the diversity of the training data, which helped the model learn more complex and variable features, enhancing its adaptability to new data. Enhanced Robustness: By introducing various disruptions in the training data, the G group’s model was forced to learn more robust feature representations, reducing sensitivity to specific noises or distortions, thus performing better in practical applications. Effective Error Identification and Correction: The combined disruptions might also help the model more effectively identify and correct errors during the self-training process, especially as the accumulated knowledge through iterations could aid the model in better understanding the true structure of the data.

In conclusion, by introducing combined disruptions during the iterative self-training process, the G group demonstrated superior performance compared to the control group (T group). This approach not only increased data diversity and model robustness but also facilitated continuous improvement throughout the iterative process. Therefore, the strategy of using a Disrupted Student model offers significant advantages in improving the iterative training of image generation for diffusion models, which is particularly suitable for complex tasks requiring high generalizability and robustness.

## 4. Discussion

### 4.1. Introduction of the Noise Student in a Cyclic Adversarial Structure

A major innovation of this paper is incorporating the DDPM diffusion generation model and YOLO detection model into a mutually adversarial iterative loop process and adding the Disrupted Student into the network to assist with filtering. This significantly reduces human resource requirements and simultaneously improves the model’s performance. Compared to models that do not perform filtering, this approach prevents overfitting and enhances the model’s robustness.

The reason for introducing the Disrupted Student is that in the actual process of iterative training, relying solely on the trained YOLO detection model for image filtering could still leave a large number of inappropriate side-scan sonar shipwreck images unremoved. These incorrect images entering the next round of iterative loop training would lead to compounding errors, thereby affecting the performance metrics of the final trained model. In previous studies, avoiding this phenomenon mostly involved manually filtering images, which is time-consuming and labor-intensive. The Disrupted Student method can greatly save on human resource requirements while preventing the selection of incorrect images.

### 4.2. Manual Selection Comparison and Similar Methods Comparison

To further verify the effectiveness of the Disrupted Student model, we introduced manual selection for comparison. Different from the training procedure described in this paper, after each round of image filtering by the YOLO detection model, the Noise Student module is omitted, and manual selection is used to remove images that do not fit the style of side-scan sonar shipwrecks. The same four iterations were conducted, and the relevant performance metrics after iterative training are shown in Table 8 (the manual group is Group B).

By comparing the performance of the manual selection group (Group B) and the Disrupted Student model group (Group G), it is observed that the performance metrics (precision, recall, and mAP) of both groups are very close after four iterations of training. This demonstrates that the Disrupted Student model can achieve a level of performance similar to manual selection through automation, without the need for human intervention. Although manual selection performed slightly better in some iterations, the Disrupted Student model showed significant competitiveness, especially in scenarios involving large-scale data or the need for rapid iteration, where its automation advantage is particularly prominent.

Detailed Analysis:

Precision (Accuracy): Throughout all iterations, the precision accuracy of Group G was very close to that of Group B, with Group G even slightly outperforming Group B in the first round. This indicates that the Disrupted Student model’s accuracy in predicting new data is nearly equivalent to manual selection, demonstrating a high capability for recognition. Recall (Recall Rate): Recall measures the model’s ability to identify relevant samples. Both groups showed similar recall values, with Group B performing slightly better in some iterations. However, Group G maintained a high recall rate, proving its efficiency in identifying truly relevant samples. mAP (Mean Average Precision): mAP is a composite metric that considers both detection accuracy and recall. Throughout the four iterations, Group G’s mAP was almost on par with Group B’s and even performed better in the first round, emphasizing the strong overall performance of the Disrupted Student model.

Compared with the manual selection group, we can conclude that the Disrupted Student model, by closely matching the performance of manual selection, shows potential as an effective automated tool. Especially in scenarios requiring the handling of large amounts of data, it provides a solution that saves human resources and accelerates iteration speed. Although it may slightly underperform in minor performance metrics compared to manual selection, considering its automation advantages and the potential for further optimization, the Disrupted Student model represents an efficient, scalable choice. It can enhance model performance in the iterative training of diffusion models and save significant labor resources.

For side-scan sonar image diffusion generation methods, Yang et al. proposed a method combining DDIM and DPM-Solver. This approach reduces the model’s sampling time by introducing an accelerated encoder. Although it improves the generation speed, it does not enhance the quality of the images produced by the diffusion model. Because the purpose of sample augmentation is to improve downstream target recognition accuracy, we use the accuracy of the YOLO detection model as the key evaluation metric. Below are the detection comparison experiments for the original DDPM model, DDIM + DPM-Solver, and DS-SIAUG, with relevant data shown in Table 9.

The analysis indicates that the DDIM + DPM-Solver method, which does not fundamentally change the training of the diffusion model but merely accelerates the training process, yields results similar to multiple rounds of training. Therefore, the detection metrics and image quality of the original diffusion model show no significant difference. The recognition accuracy of DPM + Solver is only slightly improved by 1% compared to the unfiltered original DDPM model, indicating minimal change. In contrast, the method described in this paper employs iterative training, gradually improving the quality of generated images through multiple rounds of selection and quality control. This process allows the model to fully learn all of the features of the original side-scan sonar images, resulting in superior performance in target recognition and high-quality image generation. Consequently, it enhances the model’s performance and trains a high-quality side-scan sonar diffusion model.

### 4.3. Reasons for Improved Model Performance with the Disrupted Student Model

The addition of noise proves particularly useful primarily because side-scan sonar images inherently contain a rich variety of noise features. In actual measurement processes, diverse types of noise, such as stripe noise, Gaussian noise, and speckle noise, frequently occur. Especially when detecting underwater targets like shipwrecks, certain factors, such as the varying orientations of the ship, the specific location of the transducer, and the speed of the survey vessel, can significantly impact the accuracy of the side-scan sonar images, leading to deformations in the imagery. Against these types of disruptions, the anti-interference ability of traditional neural networks appears relatively weak, necessitating a solution that can effectively enhance model robustness.

In this context, the strategy of the Disrupted Student model becomes particularly important. The core of this approach is to simulate a “level-up” learning scenario, where the teacher model first labels the augmented dataset. During the training phase, the student model receives unaltered images; however, during the validation phase, images with added disruptions are used. This practice results in the student model often performing poorly on the disrupted validation set. When faced with feedback inconsistent with the teacher model, the student model recognizes its shortcomings and undergoes self-adjustment and optimization in subsequent iterations. This process effectively promotes a deeper analysis of the data by the student model, enhances its resistance to disruptions, and ultimately achieves performance that surpasses that of the teacher model.

On the other hand, the addition of geometric disruptions can be seen as a means of dataset augmentation. By performing operations, such as rotation, mirroring, and deformation, on the original dataset, the diversity and size of the dataset are substantially increased. This method not only enriches the content of the dataset but also helps to improve its quality and diversity, thereby enhancing detection performance and, concurrently, improving the overall model performance.

In summary, whether through simulating noise to enhance model robustness or by expanding the dataset through geometric disruptions, the Disrupted Student model provides an innovative and effective pathway to improve the precision and efficiency of processing side-scan sonar images. It also increases the degree of automation in iterative training, significantly saving on human resources.

## 5. Conclusions

Facing the challenges of scarcity, small sample size, high acquisition difficulty, and cost of side-scan sonar underwater target images, we proposed the DS-SIAUG method for augmenting side-scan sonar images and conducted data augmentation experiments focusing on shipwreck targets. Through adversarial iterative training between the DDPM diffusion generation model and the YOLO target detection model, we found that after four iterations, the model reached its optimal state in the third iteration, with a 5% improvement in detection accuracy compared to the first iteration. This significantly enhanced the quality of image generation and target detection accuracy. During the augmented data selection process, we introduced the Disrupted Student model to replace manual dataset labeling. Comparative experiments showed that the Disrupted Student model improved target recognition accuracy by 1.2% compared to unfiltered data and was only 0.2% lower than manually filtered data. Preliminary results demonstrate the effectiveness of the DS-SIAUG method, significantly reducing human effort and providing an effective solution for generating side-scan sonar images, thereby addressing the issue of underwater sample target scarcity. Regarding the poor performance of the model in generating high-resolution images, we found that the reason is due to the low resolution of the training dataset itself and the limitations of computer memory capacity. Therefore, we cannot further discuss the model’s performance at higher resolutions. We hope to improve this aspect in future research.

## Figures and Tables

**Table 1:** Addition of noise disruptions.

Noise Type	Noise Characteristics	Noise Source	Addition Intensity	Proportion of Total Images
Gaussian Noise	Probability density function follows Gaussian distribution	Thermal noise from electronic devices, sensor reading errors	Random variation between 0 and 0.5	Randomly selected for 25% of images
Stripe Noise	Appears as horizontal or vertical stripes in the image	Influenced by sensor mechanical movement and temperature changes	Vertical gradient ratio randomly varies between 0 and 0.1	Randomly selected for 25% of images
Speckle Noise	Multiplicative noise, formed by the speckle pattern from multiple small beams converging	Caused by wave coherence leading to variations in wave strength, resulting in granular noise in the image	Noise size randomly chosen between 0 and 0.1	Randomly selected for 25% of images

**Table 2:** Addition of geometric deformation disruptions.

Disruption Type	Method of Addition	Proportion of Total Images
Rotation	Random rotation at 0, 90, 180, 270 degrees	100%
Mirroring	Randomly selected	50%
Deformation	Random selection of scaling ratios at 0.5, 0.75, 1.25, 1.5	66.6%

**Table 3:** DDPM training parameter settings.

Group	Training Set Category	Batch Size	Image Size
T1	Shipwreck, plane, stone	512	128*128
T2	Shipwreck	80	256*256
T3	Shipwreck	16	512*512

**Table 4:** Experimental results of different sizes of models on FID, LPIPS, PSNR, MMD, and SSIM.

Group	Training Set Category	FID	LPIPS	PSNR	MMD	SSIM
T1	Shipwreck (128*128)	123.7	0.6860	4.7845	0.00204	0.0872
	Plane (128*128)	112.3	0.8129	3.8046	0.0049	0.08077
	Stone (128*128)	102.9	0.7789	3.5698	0.0156	0.2114
T2	Shipwreck (256*256)	150.6	0.6631	4.7634	0.0045	0.0727
T3	Shipwreck (512*512)	148.2	0.6622	5.0441	0.0039	0.05479

**Table 5:** Disruption model grouping.

Group	Noise Disruption	Geometric Deformation Disruption
C1	-	-
C2	√	-
C3	-	√
C4	√	√

**Table 6:** Results of disruption experiments.

Group	Precision	Recall	Map
C1	0.854	0.807	0.869
C2	0.859	0.832	0.892
C3	0.878	0.866	0.904
C4	0.883	0.863	0.913

**Table 7:** Results of iterative training with disrupted student.

Group	Precision	Recall	Map	Group	Precision	Recall	Map
T1	0.854	0.807	0.869	G1	0.883	0.863	0.913
T2	0.873	0.862	0.901	G2	0.891	0.906	0.931
T3	0.892	0.856	0.885	G3	0.904	0.906	0.938
T4	0.889	0.827	0.885	G4	0.896	0.9	0.939

**Table 8:** Manual iteration experiment metrics analysis.

Group	Precision	Recall	Map	Group	Precision	Recall	Map
B1	0.878	0.865	0.904	G1	0.883	0.863	0.913
B2	0.892	0.916	0.931	G2	0.891	0.906	0.931
B3	0.906	0.911	0.941	G3	0.904	0.906	0.938
B4	0.900	0.876	0.939	G4	0.896	0.9	0.939

**Table 9:** Similar methods comparison.

Network	Precision	Recall	Map
DDPM	0.851	0.847	0.899
DDIM + DPM-Solver	0.862	0.853	0.913
DS-SIAUG	0.904	0.906	0.938

## Data Availability

Data are contained within the article.
